# Supplementary material for: Wood Cellulose Nanofibers Grafted with Poly(ε-caprolactone) Catalyzed by ZnEu-MOF for Functionalization and Surface Modification of PCL Films
Source: Nanomaterials (Basel). 2023 Jun 21;13(13):1904. doi: 10.3390/nano13131904 (PMC10343636; doi:10.3390/nano13131904)
Supplement: Supplementary file 1 [file nanomaterials-13-01904-s001.zip › nanomaterials-2438408-supplementary.docx]

Supplementary Materials

Wood Cellulose Nanofibers Grafted with Poly(ε-caprolactone) Catalyzed by ZnEu-MOF for Functionalization and Surface Modification of PCL Films

Jinying Pang ^1,2^, Tanlin Jiang ^2,3^, Zhilin Ke ^1,4^, Yu Xiao ^4^, Weizhou Li ^3,5^, Shuhua Zhang ^1,4,^* and Penghu Guo ^4,^*

| **Citation:** Pang, J.; Jiang, T.; Ke, Z.; Xiao, Y.; Li, W.; Zhang, S.; Guo, P. Wood Cellulose Nanofibers Grafted with Poly(ε-caprolactone) Catalyzed by ZnEu-MOF for Functionalization and Surface Modification of PCL Films. *Nanomaterials* **2023**, *13*, 1904. https://doi.org/10.3390/ nano13131904  Academic Editor: Takuya Kitaoka  Received: 23 May 2023  Revised: 19 June 2023  Accepted: 19 June 2023  Published: 21 June 2023  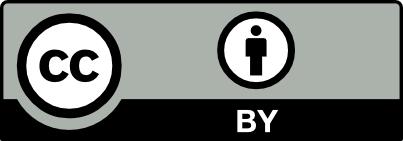  **Copyright:** © 2023 by the authors. Licensee MDPI, Basel, Switzerland. This article is an open access article distributed under the terms and conditions of the Creative Commons Attribution (CC BY) license (https://creativecommons.org/licenses/by/4.0/). |
| --- |

^1^ Guangxi Key Laboratory of Electrochemical and Magnetochemical Functional Materials,
College of Materials Science and Engineering, Guilin University of Technology, Guilin 541004, China; pangjinying@nnnu.edu.cn (J.P.); kzl@glut.edu.cn (Z.K.)

^2^ Guangxi Key Laboratory of Natural Polymer Chemistry and Physics, College of Chemistry and Materials, Nanning Normal University, Nanning 530001, China; jtl970906@126.com

^3^ College of Resources, Environment and Materials, Guangxi University, Nanning 530004, China; wzli@xmut.edu.cn (W.L.)

^4^ Key Laboratory of Petrochemical Pollution Control of Guangdong Higher Education Institutes, Guangdong Provincial Key Laboratory of Petrochemical Pollution Process and Control (College of Chemistry), Guangdong University of Petrochemical Technology, Maoming 525000, China; xiaoyu@gdupt.edu.cn

^5^ School of Materials Science and Engineering, Xiamen University of Technology, Xiamen 361024, China

***** Correspondence: zsh720108@163.com (S.Z.); penghuguo@sina.com (P.G.)

Table of Contents

S1. Typical Tables

**Table S1.** Crystal data and structures refinement for ZnEu-MOF.

**Table S2.** Selected bond lengths (Å) and angles (°) for ZnEu-MOF.

**Table S3.** Shape data of ZnEu-MOF.

**Table S4.** Cytotoxicity studies of GCL-D/PCL and 4 wt% LCF to HEK293T cells

S2. Typical Figure

S3. Supplementary Text

S3.1 Methods

S3.1.1 Synthesis of GCL

S3.1.2 In vitro cytotoxicity

S3.2 Characterization of GCL

S1. Typical Tables

**Table S1.** Crystal data and structures refinement for ZnEu-MOF.

| Complex | ZnEu-MOF |
| --- | --- |
| Formula | C_33.61_H_24.23_N_6_O_14_EuZn |
| *Fw* | 953.55 |
| Crystal system | Monoclinic |
| Space group | *C*2/c |
| *a* (Å) | 22.6521 (13) |
| *b* (Å) | 21.1226 (11) |
| *c* (Å) | 24.9222 (13) |
| *α* (°) | 90 |
| *β* (°) | 112.819 (2) |
| *γ* (°) | 90 |
| *V* (Å^3^) | 10991.3 (10) |
| *F*(000) | 3783.3 |
| *Z* | 8 |
| *D*c (g m^–3^) | 1.153 |
| *μ* (mm^–1^) | 1.618 |
| *θ* range (°) | 3.08-25.01 |
| Ref. meas. / indep. | 100534/9573 |
| Obs. ref.[*I* > 2*σ* (*I*)] | 8518 |
| *R*_int_ | 0.0562 |
| *R*_1_ [*I* ≥ 2*σ* (*I*)] ^a^ | 0.0454 |
| *ωR*_2_(all data)^b^ | 0.1278 |
| Goof | 0.997 |

^a^ *R*_1_ = Σ||*F*_o_| – |*F*_c_||/Σ|*F*_o_|. ^b^ *wR*_2_ =[Σ*w*(|*F*_o_^2^|–|*F*_c_^2^|)^2^/Σ*w*(|*F*_o_^2^|)^2^]^1/2.^

**Table S2.** Selected bond lengths (Å) and angles (°) for ZnEu-MOF.

| Eu1—O5’ | 2.362(9) | Eu1—O13^ii^ | 2.484(3) |
| --- | --- | --- | --- |
| Eu1—O10 | 2.358(3) | Eu1—O12^ii^ | 2.570(4) |
| Eu1—O1 | 2.401(4) | Zn1—N6'^iii^ | 2.00(4) |
| Eu1—O6 | 2.407(4) | Zn1—N6^iii^ | 2.02(7) |
| Eu1—O8^i^ | 2.471(4) | Zn1—O2 | 1.924(5) |
| Eu1—O9^i^  Eu1—O5  Eu1—N4^iv^ | 2.476(3)  2.65(3)  2.54(4) | Zn1—O7  Zn1—O11  Eu1—N4’^iv^ | 1.929(4)  1.933(4)  2.548(17) |
| O7—Zn1—O2 | 121.7(2) | O5’—Eu1—O10 | 85.9(3) |
| O2—Zn1—O11  O7—Zn1—O11  O2—Zn1—N6’^iii^  O7—Zn1—N6’^iii^  O11—Zn1—N6’^iii^  O2—Zn1—N6^iii^  O7—Zn1—N6^iii^  O11—Zn1—N6^iii^  O6^i^—Eu1—O8  O10—Eu1—O9^i^ | 109.7(2)  121.2(2)  106.3(13)  95.7(9)  95.6(4)  94(2)  103.9(17)  99.2(5)  107.77(17)  153.07(15) | O10—Eu1—O1  O5’—Eu1—O1  O10—Eu1—O6  O5’—Eu1—O6  O1—Eu1—O6  O10—Eu1—O8^i^  O5’—Eu1—O8^i^  O1—Eu1—O8^i^  O5’—Eu1—O9^i^  O1—Eu1—O9^i^ | 78.90(17)  141.0(3)  79.88(15)  71.4(3)  70.7(2)  146.66(15)  127.4(3)  73.63(15)  78.9(3)  99.22(18) |
| O6—Eu1—O9^i^ | 74.27(14) | O8^i^—Eu1—O9^i^ | 52.57(12) |
| O10—Eu1—O13^ii^ | 123.26(12) | O5’—Eu1—O13^ii^ | 77.3(3) |
| O1—Eu1—O13^ii^ | 140.40(16) | O6—Eu1—O13^ii^ | 139.45(16) |
| O8^i^—Eu1—O13^ii^ | 72.15(13) | O9^i^—Eu1—O13^ii^ | 74.97(14) |
| O10—Eu1—O12^ii^ | 72.11(12) | O1—Eu1—O12^ii^ | 136.46(18) |
| O6—Eu1—O12^ii^  O8^i^—Eu1—O12^ii^  O13^ii^—Eu1—O12^ii^  O6—Eu1—O5  O8^i^—Eu1—O5  O13^ii^—Eu1—O5  O10—Eu1—N4^iv^  O1—Eu1—N4^iv^  O8^i^—Eu1—N4^iv^  O13^ii^—Eu1—N4^iv^  O5’—Eu1—N4’^iv^  O6—Eu1—N4’^iv^  O9^i^—Eu1—N4’^iv^  O12^ii^—Eu1—N4’^iv^  O5—Eu1—N4^iv^ | 132.18(18)  117.04(13)  51.23(11)  68.7(7)  121.0(7)  76.9(7)  83(2)  79(3)  74(2)  72(3)  142.6(16)  138.0(14)  125.0(13)  73.9(16)  139(2) | O5’—Eu1—O12^ii^  O9^i^—Eu1—O12^ii^  O10—Eu1—O5  O1—Eu1—O5  O9^i^—Eu1—O5  O12^ii^—Eu1—O5  O5’—Eu1—N4^iv^  O6—Eu1—N4^iv^  O9^i^—Eu1—N4^iv^  O10—Eu1—N4’^iv^  O1—Eu1—N4’^iv^  O8^i^—Eu1—N4’^iv^  O13^ii^—Eu1—N4’^iv^  O12^ii^—Eu1—N4^iv^  O5—Eu1—N4’^iv^ | 68.8(3)  121.14(13)  92.2(7)  139.4(7)  71.6(7)  74.4(7)  134(2)  148(3)  123(2)  79.9(12)  69.5(15)  73.0(13)  81.9(14)  66(2)  148.2(17) |

Symmetry codes: (i) 0.5+*x*, 0.5+*y*, *z*; (ii) *x*, -*y*, 0.5+*z*; (iii) 1-*x*, -*y*, 1.5+*z*; (iv) 0.5-*x*, 1.5+*y*, 1.5-*z*.

**Table S3.** shape data of ZnEu-MOF.

| Complex | Metal | Vertices | Code | Label | Shape | Energy |
| --- | --- | --- | --- | --- | --- | --- |
| ZnEu-MOF | Eu1 | 9 | 1 | EP-9 | Enneagon | 35.765 |
|  |  |  | 2 | OPY-9 | Octagonal | 23.487 |
|  |  |  | 3 | HBPY-9 | Heptagonal bipyramid | 19.292 |
|  |  |  | 4 | JTC-9 | Johnson triangular cupola | 14.770 |
|  |  |  | 5 | JCCU-9 | Capped cube | 10.482 |
|  |  |  | 6 | CCU-9 | Spherical-relaxed capped cube | 9.588 |
|  |  |  | 7 | JCSAPR-9 | Capped square antiprism | 1.808 |
|  |  |  | 8 | CSAPR-9 | Spherical  capped square antiprism | 1.008 |
|  |  |  | 9 | JTCTPR-9 | Tricapped trigonal prism | 3.091 |
|  |  |  | 10 | TCTPR-9 | Spherical tricapped trigonal  prism | 1.771 |
|  |  |  | 11 | JTDIC-9 | Tridiminished icosahedron | 11.888 |
|  |  |  | 12 | HH-9 | Hula-hoop | 11.813 |
|  |  |  | 13 | MFF-9 | Muffin | 1.425 |
|  | Zn1 | 4 | 1 | SP-4 | Square | 51.596 |
|  |  |  | 2 | T-4 | Tetrahedron | 38.045 |
|  |  |  | 3 | SS-4 | Seesaw | 43.181 |

**Table S4.** Cytotoxicity studies of GCL-D/PCL and 4 wt% LCF to HEK293T cells.

| Concentration (µg/mL) | Cell viability (%) | | | |
| --- | --- | --- | --- | --- |
|  | GCL-D/PCL  (24 h) | 4 wt% LCF  (24 h) | GCL-D/PCL  (48 h) | 4 wt% LCF  (48 h) |
| 0 | 100.000±0.867 | 100.000±0.576 | 100.000±6.236 | 100.000±10.622 |
| 10 | 103.178±1.404 | 112.826±3.161 | 106.040±9.394 | 117.358±10.657 |
| 50 | 93.834±0.649 | 110.346±1.109 | 101.013±7.248 | 113.829±8.289 |
| 100 | 87.445±3.185 | 106.407±4.132 | 89.507±9.073 | 106.477±10.067 |
| 500 | 78.400±1.809 | 98.201±5.115 | 83.568±1.694 | 99.863±5.887 |
| 1000 | 61.143±0.719 | 85.731±2.590 | 68.204±2.025 | 87.608±1.087 |

**S2. Typical Figure**





**Figure S1.** PXRD of TG final residual material with ZnEu-MOF and card of ZnO and Eu_2_O_3_.


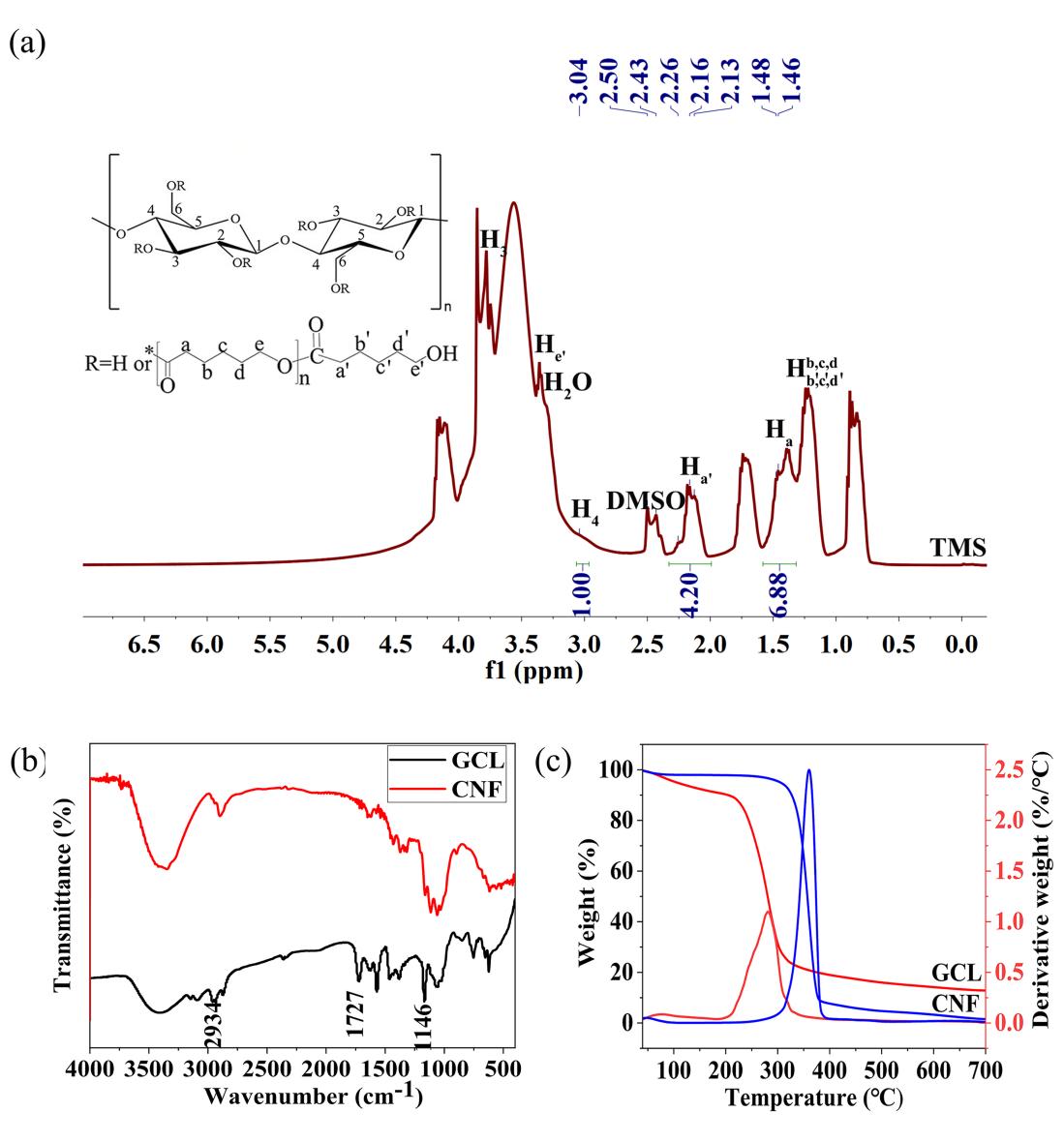


**Figure S2.** (a) ^1^H-NMR spectrum of GCL in DMSO‑*d_6_*; (b) Infrared spectrum of GCL and CNF; (c) The TG and DTG curves of GCL and CNF.

S3. Supplementary Text

S3.1 Methods

S3.1.1 Synthesis of GCL

According to our orthogonal experiments, the optimal reaction conditions were determined to be an [AGU]/[ε-CL] (AGU =anhydroglucose unit) ratio of 1:30, a catalyst loading of 2 wt %, a reaction temperature of 120 °C, and a reaction time of 12 h. According to a reported procedure [1, 2]. The synthesis was conducted under strictly anhydrous conditions with the protection of nitrogen. A mixture of 0.25 g wood cellulose nanofibers (CNF) and 5.00 g [Bmim]Cl was added in a 100 mL dried three-neck flask and kept at 80 °C under vigorous stirring for 2 h to obtain a homogeneous solution. The monomers of ε-caprolactone and ZnEu-MOF catalyst were added to the mixture. At the end of the reaction, the product was poured into a 500 mL beaker and 250 mL anhydrous ethanol was poured into the beaker. The beaker was sealed and allowed to stand for 12 h and filtered to remove the ionic liquid and unreacted ε-CL monomer. Then 250 mL dichloromethane was poured into soak for 1 h, and the GCL was washed to remove the polycaprolactone (PCL) produced by ε-caprolactone self-homolysis during the graft reaction. The process was repeated three times. The copolymer was dried at 25 °C for 48 h.

S3.1.2 In vitro cytotoxicity

Cells in good growth state were prepared into cell suspension with a certain concentration, and 100μL per well was added into 96-well cell plates and cultured overnight in an incubator. The GCL-D/PCL and 4 wt% LCF were dissolved using DMSO and the concentration was 40 mg/mL, then, it was diluted to 4 mg/mL by a conditioning medium, the concentrations of working solutions with 0, 10, 50, 100, 500, 1000 μg/mL were diluted by 4 mg/mL drug solution. The culture medium was taken out from the culture plate, and 100 μL working liquid of different concentrations was remixed and hatched for 24 h and 48 h, severally. And 100 μL medium containing 10% cell counting Kit-8 was added, and the solution was incubated for 1 h. The absorption value at 450 nm was investigated using Thermo Multiskan SKY.

S3.2 Characterization of GCL

The ^1^H-NMR spectrums of GCL are shown in Fig. S2a. The peak of chemical shift *δ*= 4.0 ppm corresponded to methylene hydrogen (H_e_) in the structure of repeating unit −CH_2_O− in the PCL chain, and the peak of *δ*= 3.37 ppm corresponded to hydrogen (He') on −CH_2_OH− at the end of the polycaprolactone chain. Chemical shift *δ* = 2.13-2.26 ppm corresponded to hydrogen (Ha') on −COCH_2_− at the end of the PCL chain, while *δ* = 1.46-1.48 ppm corresponded to hydrogen (Ha) on the repeat unit −COCH_2_− in the PCL chain. Meanwhile, in the range of *δ* =1.21-1.40 ppm, the hydrogen corresponded to methylene at repeat units b, d and c in the polycaprolactone chain was obtained. The signal peak of hydrogen H_4_ on the glucose unit of nanocellulosic was about 3.04 ppm, and the hydrogen signal peaks of other glucose units were in the range of *δ*=3.2 -5.6 ppm[3]. Some other mixed peaks in the Figure indicated that the complex catalyst was embedded in the GCL. These results of FT-IR and ^1^H NMR confirmed the successful grafted with of PCL onto the surface of CNF.

The grafting ratio could be calculated by integrating the correlation peaks of the ^1^H NMR spectra of the GCL. The grafting ratio intuitively reflected the catalytic activity of the MOF in the ring-opening polymerization of ε-caprolactone. The molar substitution degree (*MS*) and grafting ratio (*W_PCL_*) of PCL were calculated as follows:

$$MS=I_{\left( a+a^{'} \right)}/2H_{4} (S1)$$

$W_{PCL}=114MS/(162+114MS)$ (S2)

114 g/mol was the molar mass of ε-caprolactone and 162 g/mol was the molar mass of nanocellulose glucose unit. the grafting ratio of GCL was 79.38%.

FT-IR results of CNF and GCL are plotted in Fig. S2b. Three new characteristic peaks appear in the infrared spectrum of GCL compared with the CNF. The characteristic peaks at the wave number of 2934 cm^-1^ and 1146 cm^-1^ corresponded to the C−H stretching and bending vibration peaks of−CH_3_ and −CH_2_ on the PCL adipose chain. The absorption peak at 1727 cm^-1^ was attributed to the stretching vibration of the C=O group of the polycaprolactone chain[3]. According to these characteristic peaks of the GCL infrared spectrum, it can be preliminatively judged that the PCL was successfully grafted to cellulose nanofibers the chain under the catalytic action of the ZnEu-MOF.

The TGA curve of CNF and GCL were shown in the in Fig. S2c, there was a small range of decomposition peak in the range of 25-100 ℃, because CNF and GCL had a certain adsorption effect on water in the air, and the absorbed water in this temperature range escapes. The maximum decomposition peak of CNF occurred at about 350 ℃, while the maximum decomposition peak of the GCL was between 250 and 300 ℃. Because CNF grafted with poly(ε-caprolactone), the hydrogen bond network connecting the molecular chains of CNF was destroyed and the thermal stability decreased, which was consistent with the results reported in the literature[2]. Several strong decomposition peaks appeared in the process of the GCL decomposition. These extra peaks could be attributed to the decomposition of the ZnEu-MOF catalyst in the GCL. When the heated temperature reached 700 ℃, the CNF was completely decomposed, and the GCL still had 12.8 % of the mass remaining, which also indicated that there were a small amount of catalysts remaining in the GCL, and these residual substances were metal oxides produced by the decomposition and oxidation of the ZnEu-MOF.

References

1. Guo, Y.; Wang, X.; Shen, Z.; Shu, X.; Sun, R. Preparation of Cellulose-Graft-Poly(ε-caprolactone) Nanomicelles by Homogeneous Rop in Ionic Liquid. *Carbohydr. Polym.* **2013**, *921*, 77–83.
2. 2Zuppolini, S.; Maya, I.C.; Diodato, L.; Guarino, V.; Borriello, A.; Ambrosio, L. Self-Associating Cellulose-Graft-Poly(ε-caprolactone) to Design Nanoparticles for Drug Release. *Mater. Sci. Eng. C* **2020**, *108*, 110385.
3. Pang, J.; Gao, Q.; Yin, L.; Zhang, S. Synthesis and Catalytic Performance of Banana Cellulose Nanofibres Grafted with Poly(ε-caprolactone) in a Novel Two-Dimensional Zinc(II) Metal-Organic Framework. *Int. J. Biol. Macromol.* **2023**, *224*, 568–577.
